# DMRT2 Interacts With FXR and Improves Insulin Resistance in Adipocytes and a Mouse Model

**DOI:** 10.3389/fendo.2021.723623

**Published:** 2022-02-17

**Authors:** Jing Tao, Xiao-Lin Yu, Yu-Juan Yuan, Xin Shen, Jun Liu, Pei-Pei Gu, Zhao Wang, Yi-Tong Ma, Guo-Qing Li

**Affiliations:** ^1^ Department of Cardiology, People’s Hospital of Xinjiang Uygur Autonomous Region, Urumqi, China; ^2^ Department of Cardiology, The First Affiliated Hospital of Xinjiang Medical University, Urumqi, China; ^3^ Graduate School of Xinjiang Medical University, Urumqi, China

**Keywords:** obesity, insulin resistance (IR), double-sex and mab-3-related transcription factor 2 (DMRT2), farnesoid X receptor (FXR), 3T3-L1 adipocytes

## Abstract

Insulin resistance (IR) plays a critical role in cardiovascular diseases and metabolic diseases. In this study, we identified the downregulation of DMRT2 in adipose tissues from insulin-resistant subjects through bioinformatics analysis and in an insulin-resistant mouse model through experimental analysis. DMRT2 overexpression significantly attenuated HDF-induced insulin resistance and inflammation in mice. Moreover, in control and insulin-resistant differentiated mouse 3T3-L1 adipocytes, DMRT2 overexpression attenuated but DMRT2 knockdown enhanced the insulin resistance of 3T3-L1 adipocytes. DMRT2 interacted with FXR and positively regulated FXR level and transcription activity. In both control and insulin-resistant differentiated mouse 3T3-L1 adipocytes, FXR knockdown enhanced the insulin resistance and attenuated the effects of DMRT2 overexpression upon 3T3-L1 adipocyte insulin resistance. In conclusion, we identify the downregulation of DMRT2 in the insulin-resistant mouse model and cell model. DMRT2 interacts with FXR and improves insulin resistance in adipocytes.

## Introduction

Obesity is widely prevalent in the world. Currently, 39% of the total population are overweight and 13% are obese (1, 2). In obesity, adipose tissue remodeling is accompanied by extracellular matrix remodeling, inflammatory cell infiltration, abnormal adipokines, and free fatty acid secretion, which causes chronic inflammation, in turn leading to insulin resistance and ultimately to hypertension, atherosclerosis, coronary artery disease, and cardiovascular diseases (3).

Insulin resistance (IR) is a state of decreased responsiveness of target tissues to normal circulating levels of insulin or impaired ability of circulating or injected insulin to decrease blood glucose levels at the whole-organism level (4), resulting in the biological production of insulin and a pathophysiological state of secondary hyperinsulinemia (5, 6). Studies have shown that abnormal body fat distribution or obesity will cause insulin resistance (7, 8). For example, Radin’s team injected free fatty acids in rats to cause insulin resistance in their skeletal muscles, which was partially improved in TLR4 knockout rats (9). They also indicated that high free fatty acid might be the leading cause of insulin resistance due to obesity (9). Therefore, understanding the mechanism of the occurrence and development of IR would provide new strategies to relieve IR and more effective therapies for metabolic diseases.

Several studies have analyzed differentially expressed genes in adipose tissues between obese and normal subjects (10–12). Nono Nankam et al. (13) found that the expression of 15 genes differed between these fat depots at baseline. These genes were found to be primarily linked to embryonic development (including *HOXA5*, *DMRT2*, *HOXB8*, *IRX5*, *IRX2*) and modulation of anatomical morphogenesis (including *DMRT2*, *DMRT3*, *HOXA5*, *RSPO3*). Unlike the homeobox family, few studies have been conducted on DMRT (double-sex and mab-3-related transcription factor) family in obesity and body fat distribution. In addition to being mainly associated with sexual differentiation and gonad development in the process of embryogenesis of numerous species (14–16), DMRT gene expression in subcutaneous abdominal adipose tissue of individuals with obesity has also been reported (16). Nono Nankam et al. (13) identified DMRT2 upregulation in abdominal subcutaneous adipose tissue vs. gluteal subcutaneous adipose tissue at baseline and post-exercise training, which is consistent with the study by Passaro et al. (17) in healthy, resting men. Therefore, *DMRT2* may be considered as a new candidate gene that participates in the regulation of central lipid accumulation. Notably, a recent study on 493 cases of sedentary adults found that DMRT2 is closely related to the intrinsic cardiopulmonary health of adults (17). Linc-DMRT2, which is transcribed from the *DMRT2* gene, is decreased within the adipose tissues of patients with inflammation-induced cardiometabolic diseases, and the level of linc-DMRT2 is closely related to lipopolysaccharide (experimental endotoxemia) in the blood (18). As mentioned above, DMRT2 exhibits a tight association with adipocytes and the pathogenesis of cardiovascular diseases; however, its specific role and mechanism in adipocyte IR remain unclear.

In the present study, we established a mouse model of insulin resistance and verified this model by glucose tolerance test (GTT), insulin tolerance test (ITT), body weight and epididymal fat weight examination, and histopathological examination. The expression of DMRT2 was examined in a mouse obese model. DMRT2 overexpression was achieved in the mouse model and the effects of DMRT2 on mice insulin resistance were examined. Next, mouse preadipocytes 3T3-L1 were induced to mature adipocytes and an insulin-resistant cell model was established based on differentiated 3T3-L1 adipocytes. The effects of DMRT2 overexpression or knockdown on insulin-resistant cell model were examined. For further molecular mechanism, protein–protein interaction analysis was performed and FXR (NR1H4, bile acid receptor) was predicted to interact with DMRT2. The FXR is a nuclear receptor whose activation leads to alterations in pathways involved in energy metabolism (19). FXR can increase the expression of small heterodimer partner (SHP) in the liver tissue, thereby inhibiting cytochrome P450 7A1 (CYP7A1) expression, regulating bile salt export pump (BSEP) expression, and improving hepatic bile acid metabolism (20, 21). As a main insulin-responsive glucose transporter, glucose transporter 4 (GLUT4) could be upregulated by FXR *via* FXR response element in GLUT4 promoter (22). The predicted interaction was examined and the dynamic effects of DMRT2 and FXR on the insulin-resistant cell model were examined.

## Materials and Methods

### Mice and Animal Model of Insulin Resistance

C57BL/6 mice (specific pathogen-free, male, 6–8 weeks old) were obtained from SJA Laboratory Animal Co., Ltd. (Changsha, China). All mice can eat and drink freely and were kept in a temperature-controlled colony room with a 12-h/12-h light/dark cycle. All experimental procedures were in compliance with the National Institutes of Health guidelines and were approved by the local Animal Care and Use Committee of the People’s Hospital of Xinjiang Uygur Autonomous Region. The mice were divided into four groups: the normal control group in which mice took a normal diet (10% of calories in fat) for 12 weeks, the high-fat diet (HFD) group in which mice took a high-fat diet (60% of calories in fat) for 12 weeks, the high-fat diet + lv-vector group in which mice took a high-fat diet and received tail vein injection of control empty lentivirus once a week for 12 weeks (100 μl, 10^9^ TU/ml), and the high-fat diet + lv-DMRT2 OE group in which mice took a high-fat diet and received tail vein injection of lentivirus overexpressing DMRT2 once a week for 12 weeks (100 μl, 10^9^ TU/ml).

Two weeks before sacrifice, the GTT was performed. Three weeks before sacrifice, the ITT was performed. For the GTT experiment, the mice were fasted for 16 h and given 1 g/kg glucose by intraperitoneal injection. The blood glucose concentration was measured at 0, 30, 60, 90, and 120 min. For the ITT experiment, the mice were fasted for 6 h, and 0.35 U/kg insulin was given by intraperitoneal injection. The blood glucose concentration was measured at 0, 30, 60, 90, and 120 min. For the GTT and ITT experiments, the area under the curve (AUC) of each group was calculated. Mouse body weight and epididymal fat weight were recorded.

### Quantitative Real-Time PCR

Total RNA was extracted from target tissues or cells using RNeasy micro kit (Cat. No./ID: 74004, Qiagen, Hilden, Germany). The RNA concentration and purity were determined by the Nano-500 microspectrophotometer (Allsheng, China). The ratio of A260/280 absorption value of RNA between 1.8 and 2.0 is indicative of highly purified RNA. RNA reverse transcription was performed using High-Capacity cDNA Reverse Transcription Kit (Cat. No. 4368814, Applied Biosystems, Foster City, CA, USA). The levels of transcripts were measured by quantitative real-time PCR on an ABI Prism 7900 HT (Applied Biosystems). *β-Actin* was used as a housekeeping gene. The relative expression of each target gene relative to the control group was calculated with the 2−ΔΔCt method. The primers are listed in **Table S1**.

### ELISA Assays for TNF-α and IL-6

The levels of TNF-α and IL-6 in cell culture medium and epididymal adipose tissues were determined by mouse TNF-α ELISA kit (CSB-E04741m, CUSABIO) and mouse IL-6 ELISA kits (ab100713, Abcam) according to the instructions of the manufacturers.

### Histological Analyses by H&E and Immunohistochemical Staining

The isolated epididymal adipose tissues and liver tissues were fixed in 4% paraformaldehyde and paraffin-embedded. Then, the tissue samples were cut into 4-μm-thick sections and deparaffinized, rehydrated with graded alcohols, and finally subjected to H&E staining and examination by light microscopy. Immunohistochemistry was performed in the sections with anti-DMRT2 (PA5-41694, Thermo, USA), anti-F4/80 (29414-1-AP, Proteintech, USA), anti-UCP-1 (23673-1-AP, Proteintech), BSEP (18990-1-AP, Proteintech), SHP (NR0B2, Boster, China), and Cyp7A1 (D161909-0025, Sangon Biotech, China) as previously described (23). Sections were visualized by Super Vision two-step kit (Boster, China). Results were observed and photographed by a light microscope.

### Cell Lineage, Cell Culture, and Adipogenesis Induction

Mouse preadipocyte 3T3-L1 (CL-173) cell line was obtained from the ATCC (Manassas, VA, USA) and cultured in Dulbecco’s modified Eagle’s medium (DMEM; Sigma-Aldrich) supplemented with 10% FBS (Invitrogen, Carlsbad, CA, USA). Cells were cultured at 37°C in 5% CO_2_.

For the differentiation of 3T3-L1 preadipocytes into mature adipocytes, 3T3-L1 cells were incubated in a medium containing 0.5 mM IBMX, 10 µg/ml insulin, and 0.25 mM DEX in DMEM containing 10% FBS for initial differentiation. After 2 days, the differentiation medium was changed to a maintenance medium (10 µg/ml insulin in DMEM containing 10% FBS) for another 6~8 days. After this period, in which at least 95% of the cells had accumulated fat droplets, mature adipocytes were used for further experiments.

### Oil Red O Staining

For validation of adipogenesis, the cells were fixed with 10% formalin in PBS for 1 h and washed twice with 60% isopropanol. The fixed cells were stained with oil red O solution for 30 min and washed with distilled water. After drying, the representative images of the fixed cells were taken. The staining area was quantified by ImageJ (NIH, USA).

### Induction of Insulin Resistance of the Cell Model Based on 3T3-L1 Cells

As previously reported (24, 25), 3T3-L1 preadipocytes were differentiated into adipocytes and induced insulin resistance. The cells were cultured in the aforementioned adipogenesis induction medium for 10 days. In order to establish an insulin resistance model, mature adipocytes 3T3-L1 were treated with 1 μM dexamethasone for 72 h. The insulin resistance of 3T3-L1 adipocytes following dexamethasone treatment was confirmed by an insulin-stimulated glucose uptake assay.

### Glucose Uptake Assay

Glucose uptake was assayed using the glucose uptake colorimetric assay kit (Sigma-Aldrich). Briefly, differentiated 3T3-L1 adipocytes were serum-starved for 8 h. Then, the adipocytes were washed with PBS (pH 7.4) and then incubated for 30 min in Krebs–Ringer bicarbonate HEPES buffer (KRBH) with insulin (1 μM), followed by the addition of 2-DG (final concentration 10 mM) and incubated for 20 min at 37°C. The cell lysate was neutralized by the addition of neutralization buffer and centrifuged. The remaining lysate was then diluted with assay buffer. Glucose uptake was detected by an enzymatic reaction to oxidize 2-DG6P to generate NADPH, which was then amplified and utilized by glutathione reductase to produce glutathione. Glutathione produces TNB by reacting with the substrate DNTB added to the reaction. TNB was then detected at 412 nm by a spectrophotometer (Bio-Rad). The glucose uptake values were normalized to protein levels, which were determined using a BCA protein assay (Beyotime, China). Each experiment was performed with three technical replicates. The results were present as fold change (treatment/control) by setting the control as 100%.

### Triglyceride Assay

The cellular triglyceride content was determined by the triglyceride content assay kit (BC0625, Solarbio, China). Differentiated 3T3-L1 adipocytes were lysed by reagent I and sonicated for 1 min. After centrifugation (8,000*g* for 10 min at 4°C), the supernatant was collected for triglyceride determination according to the instruction of the manufacturer.

### Immunofluorescence Staining

Immunofluorescence stains were performed on 4-μm sections of formalin-fixed, paraffin-embedded tissue. Antigen retrieval was induced by heating the samples to 95°C for 30 min in citrate buffer. After permeabilizing and blocking, we incubated the sections with anti-GLUT4 (66846-1-1G, Proteintech, Wuhan, China), DMRT2 (PA5-41694, Thermo, USA), and FXR (25055-1-AP, Proteintech) overnight at 4°C and then used the secondary fluorescently labeled antibody (FITC- or Cy3-labeled, Beyotime). Fluorescent images were taken with a fluorescence microscope.

### Protein Levels Determined by Immunoblotting

For the determination of GLUT4, Akt, p-Akt, DMRT2, and FXR protein levels, cells or tissues were lysed in RIPA buffer with 1% PMSF for protein extraction following the methods described before. For membrane protein isolation, the Membrane and Cytosol Protein Extraction Kit (Beyotime, China) was used. A bicinchoninic acid protein assay kit (Pierce; Thermo Fisher Scientific, Waltham, MA, USA) was used for the detection of protein concentration. The protein sample (50 μg) was fractionated using an SDS-polyacrylamide gel (10%–15%) and electrophoretically transferred to polyvinylidene fluoride (PVDF) membranes. Then, the membranes were probed with proper primary antibodies listed as follows: GLUT4 (66846-1-1G, Proteintech), Akt (ab8805, Abcam), p-Akt (ab38449, Abcam), DMRT2 (PA5-41694, Thermo), FXR (25055-1-AP, Proteintech), UCP-1 (23673-1-AP, Proteintech), BSEP (18990-1-AP, Proteintech), SHP (NR0B2, Boster), Cyp7A1 (D161909-0025, Sangon Biotech), GAPDH (T0004, Affinity Biosciences), and α-tubulin (11224-1-AP, Proteintech). Then, anti-mouse or anti-rabbit IgG coupled to peroxidase was used as a secondary antibody (Beyotime, China). Immunoreactivity was detected using the Odyssey Infrared Imaging System (Gene Company Limited, Hong Kong, China). The band intensity was measured by ImageJ software. The protein expression was normalized to α-tubulin or GAPDH.

### Co-Immunoprecipitation Assay

The sequences encoding DMRT2 and FXR were cloned into pcDNA3.1-Flag and pcDNA3.1-HA vectors and named Flag-DMRT2 and FXR-HA, respectively. Then, these vectors were co-transfected into 3T3-L1 adipocytes. The empty vector was co-transfected into target cells as a negative control. After transfection for 48 h, the cell proteins were harvested, and immunoblotting examined the expression of the labeled target protein in cells. For the co-immunoprecipitation assay, cell lysates were centrifuged and the supernatant was incubated with Protein G Dynabeads or Dynabeads conjugated with antibodies against Flag (Sigma-Aldrich) at 4°C overnight. The beads were washed three times with cell lysis buffer and the precipitated proteins were then subjected to electrophoresis and Western blotting for detection of Flag (ab205606, Abcam) and HA (ab9110, Abcam). For determining the endogenous interaction of DMRT2 and FXR, the untransfected 3T3-L1 adipocyte lysate underwent co-immunoprecipitation (co-IP) protocol using Dynabeads conjugated with antibodies against FXR (#72105S, Cell Signaling Tech, USA). The precipitated proteins were then subjected to electrophoresis and Western blotting for detection of DMRT2 and FXR.

### Luciferase Assay

The mouse GLUT4 promoter was cloned by PCR and cloned into psiCheck-2 vector (Promega, Madison, WI, USA) using homologous recombination methods. The reporter vector or DMRT2 overexpression vector was co-transfected in 3T3-L1 adipocytes in the presence or absence of DY268 (5 μM for 24 h, R&D Systems). Forty-eight hours after transfection, luciferase alterations were determined in a Dual-luciferase Reporter Assay System (Promega, USA).

### Data Processing and Statistical Analysis

The data from three independent experiments were expressed as the mean ± standard deviation, and statistical analysis was performed by one-way analysis of variance (ANOVA) followed by Tukey’s multiple comparison test or independent sample *t*-test with the SPSS Statistics 17.0 software. The significance level was based on the probability of *P <*0.05 and *P <*0.01.

## Results

### Increased DMRT2 Expression in Insulin-Resistant Adipose Tissues

Firstly, we analyzed differentially expressed genes in obese subjects or insulin-resistant subjects. There are 13 downregulated genes in obese subjects (both downregulated in GSE15524 and GSE12050). To further confirm the candidate factors of the 13 genes, DMRT2 was also significantly downregulated in insulin-resistant subjects. The flowchart of the selection process is presented (**Figure S1A**). According to GSE15524, DMRT2 expression was significantly downregulated in subcutaneous and omental abdominal adipose tissues from subjects with obesity compared with non-obese (**Figure S1B**). According to GSE12050, DMRT2 expression was significantly downregulated in subcutaneous adipose tissue from subjects with obesity compared with non-obese (**Figure S1C**). According to GSE15773, DMRT2 expression was significantly downregulated in insulin-resistant adipose tissues (**Figure S1C**). By using the MEM database matrix, genes positively correlated with DMRT2 expression in 1,794 sets of gene microarray data were shown (**Figure S1D**). Among these genes were glucose-6-phosphate isomerase (GPI), respiratory chain complex NADH-coenzyme Q (CoQ) reductase, 7-dehydrocholesterol reductase, and thioredoxin 2, which were all related to adipocyte metabolism, further indicating that DMRT2 could be involved in adipocyte function and insulin resistance.

### *In-Vivo* Effects of DMRT2 in Insulin-Resistant Mouse Model

**Figure 1:**
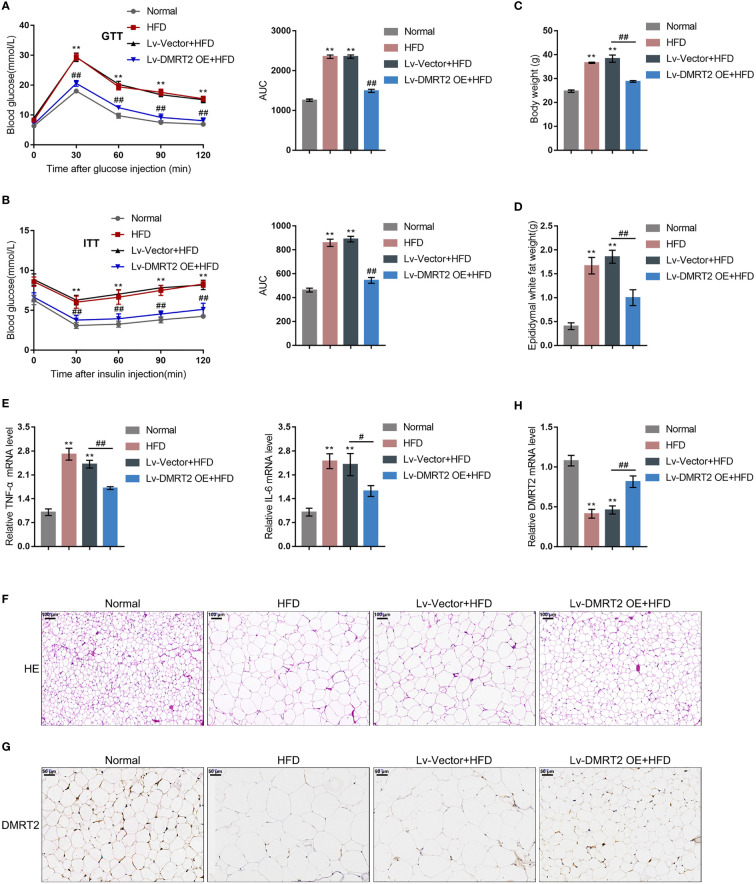
*In-vivo* effects of DMRT2 in an insulin-resistant mouse model. The insulin-resistant model was established in mice as described. DMRT2 overexpression was achieved in control or model mice by injecting lentivirus containing DMRT2 OE. **(A, B)** Glucose tolerance test (GTT) and insulin tolerance test (ITT) were performed on mice as described and the area under curve (AUC) was calculated for each group. **(C, D)** Mice body weight and epididymal fat weight were examined. **(E, H)** The mRNA expression of TNF-α, IL-6, and DMRT2 in epididymal fat tissues was examined using qRT-PCR. **(F)** The histopathological characteristics of epididymal fat tissues were examined using H&E staining. **(G)** The levels of DMRT2 in epididymal fat tissues were examined using immunohistochemical (IHC) staining. **P < 0.01, compared with the normal group; ^#^ *P* < 0.05, ^##^ *P* < 0.01, compared Lv-Vector+HFD with the Lv-DMRT2 OE+HFD group.

**Figure 2:**
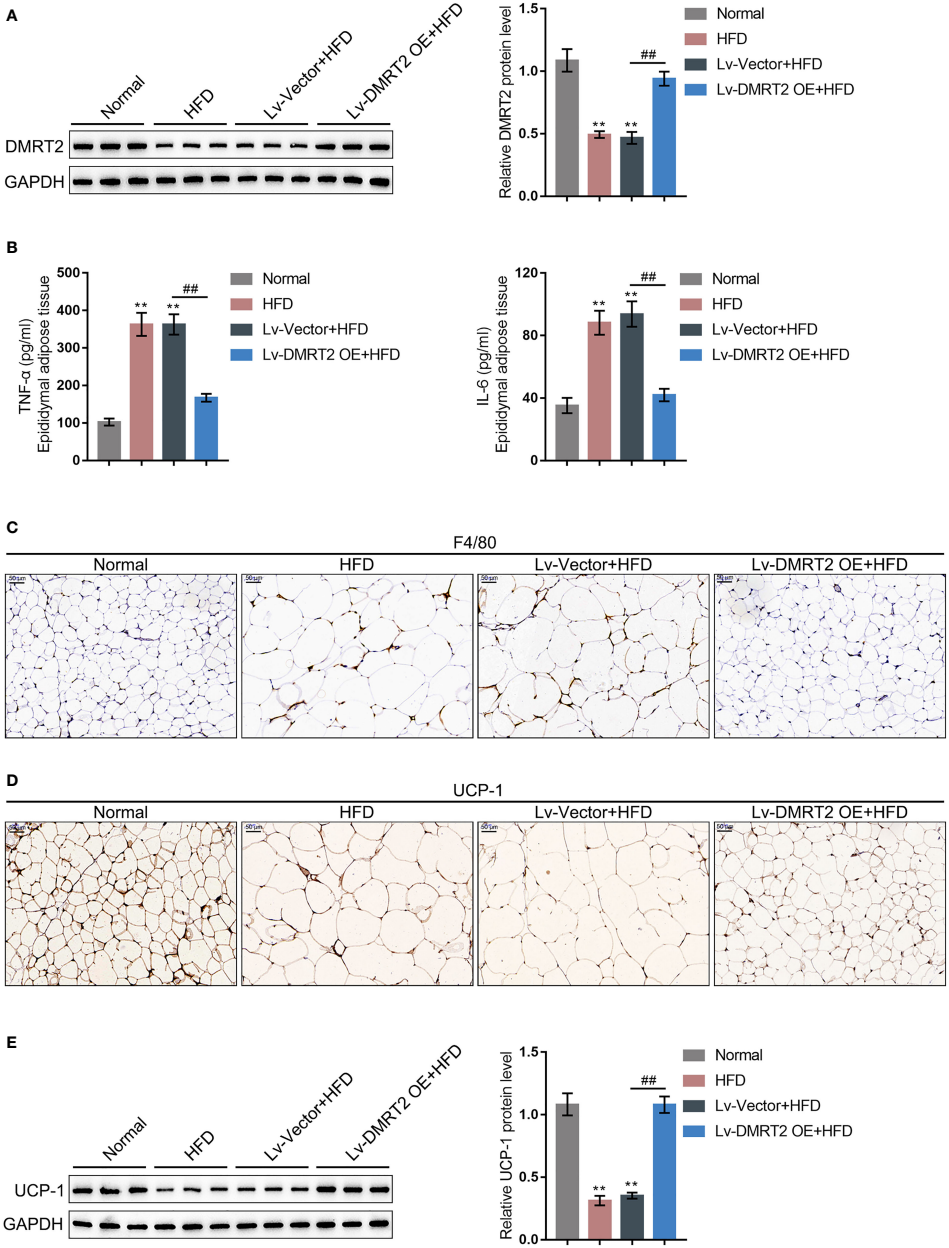
DMRT2 reduced inflammation and increased white adipose browning in the insulin-resistant mouse model. **(A)** The protein level of DMRT2 in epididymal fat tissues was examined using immunoblotting. **(B)** The protein levels of TNF-α and IL-6 in epididymal adipose tissues were examined using ELISA. **(C)** The macrophage infiltration was determined using IHC staining for F4/80. **(D, E)** The brown adipocytes in epididymal adipose tissues were determined using IHC staining and immunoblotting for UCP-1. **P < 0.01, compared with the normal group; ^##^ *P* < 0.01, compared Lv-Vector+HFD with the Lv-DMRT2 OE+HFD group.

To investigate the specific role of DMRT2 in resistance to insulin, a mouse model of insulin resistance as described was built. DMRT2 overexpression was achieved in the control or model mice by injecting lentivirus containing DMRT2 OE. To confirm the insulin-resistant model, GTT and ITT analyses were performed on mice and the AUC was calculated for each group. As shown in **Figures 1A, B**, the HFD caused impaired glucose tolerance, which showed to be dramatically weakened by DMRT2 overexpression; HFD increased insulin resistance, which was markedly rescued by DMRT2 overexpression. Consistently, HFD significantly increased but DMRT2 overexpression partially reduced mice body weight and epididymal adipose weight (**Figures 1C, D**). In epididymal fat tissues, HFD upregulated TNF-α and IL-6 mRNA expression but downregulated DMRT2 mRNA expression; DMRT2 OE partially decreased the mRNA expression levels of TNF-α and IL-6 but increased DMRT2 expression (**Figures 1E, H**). The histopathological characteristics of epididymal fat tissues examined by H&E staining further confirmed the above findings (**Figure 1F**). As shown by immunohistochemical (IHC) staining and immunoblotting, the levels of DMRT2 were decreased in epididymal fat tissues of HFD mice but partially increased by DMRT2 overexpression (**Figures 1G**, **2A**). Similarly, DMRT2 expression showed a similar trend in subcutaneous adipose tissues (**Figure S2**). With regard to inflammation, the TNF-α and IL-6 protein levels and macrophage infiltration (F4/80 positive cells) in epididymal adipose tissues were increased in HFD mice but decreased by DMRT2 overexpression (**Figures 2B, C**). White adipose tissue browning is an effective therapeutic strategy for obesity (26). Therefore, the brown adipocyte marker UCP-1 was determined in epididymal adipose tissue. As shown in **Figures 2D, E**, decreased UCP-1 was observed in HFD mice that could be increased by DMRT2 overexpression. Moreover, the expression of bile acid and lipid metabolism-related genes *CYP7A1*, *SHP*, and *BSEP* in liver tissues was also modulated by DMRT2 overexpression. *BSEP* and *SHP* levels were reduced in liver tissues of HFD mice but partially increased by DMRT2 overexpression. In contrast, *CYP7A1* level was increased in liver tissues of HFD mice but reduced by DMRT2 overexpression (**Figure S3**). Those results suggested that DMRT2 reduced the weight of body and epididymal white adipose might associate with the modulation of expression of bile acid and lipid metabolism-related genes in the liver and increased the brown adipocytes.

### DMRT2 Is Downregulated in the IR Adipocyte Model

**Figure 3:**
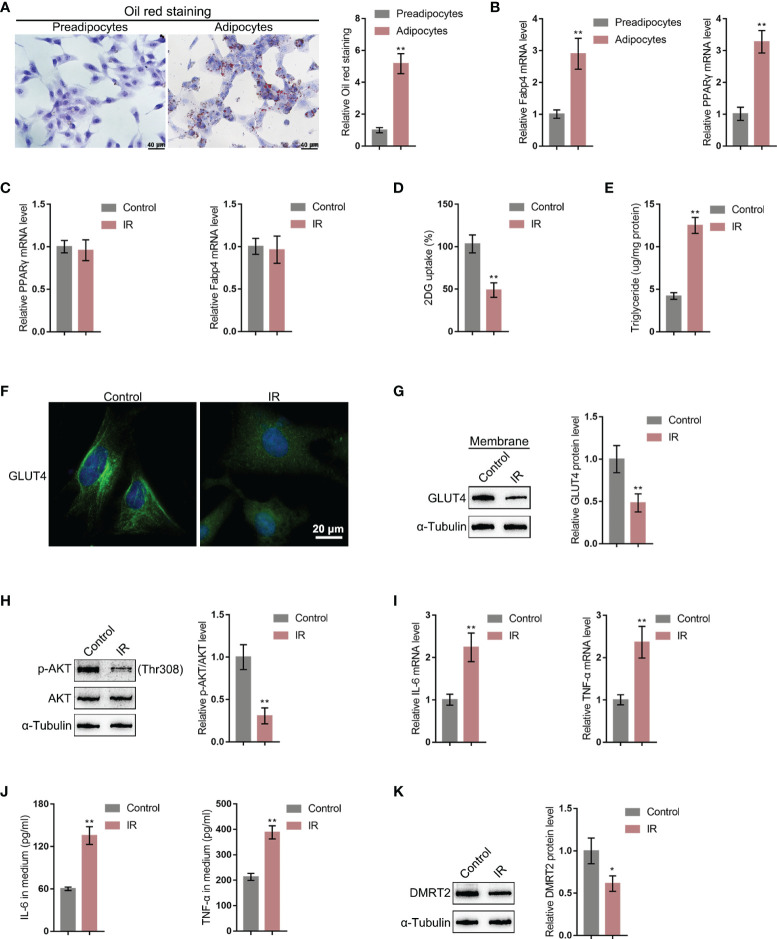
DMRT2 is downregulated in insulin-resistant adipocyte model. **(A)** 3T3-L1 preadipocytes were induced toward adipogenesis and the formation of lip droplets in preadipocytes and adipocytes was confirmed using oil O red staining. **(B)** The mRNA expression levels of Fabp4 and PPARγ in preadipocytes and adipocytes were examined using qRT-PCR. Then, the insulin-resistant adipocyte model was established in differentiated adipocytes as described and examined for Fabp4 and PPARγ mRNA levels **(C)**, glucose uptake ability **(D)**, and triglyceride content **(E)**; cellular membrane-located GLUT4 levels in control or insulin-resistant (IR) adipocytes by immunofluorescent (IF) staining **(F)**; membrane protein levels of GLUT4 in control or IR adipocytes by immunoblotting **(G)**; the protein levels of Akt and p-Akt in control or IR adipocytes by immunoblotting **(H)**; the mRNA expression of TNF-α and IL-6 in control or IR adipocytes by qRT-PCR **(I)**; the secretion levels of TNF-α and IL-6 in control or IR adipocytes by ELISA **(J)**; and the protein levels of DMRT2 in control or IR adipocytes by immunoblotting **(K)**. *P < 0.05, **P < 0.01.

To further investigate the molecular effects and mechanism of DMRT2 in resistance to insulin, the IR adipocyte model was built. Firstly, 3T3-L1 preadipocytes were induced toward adipogenesis and the formation of lip droplets in preadipocytes and adipocytes was confirmed using oil O red staining; as shown in **Figure 3A**, the formation of lip droplets was promoted in adipocytes. Then, we examined the mRNA expression of adipocyte markers, fatty acid-binding protein (Fabp4), and PPARγ using quantitative real-time PCR (qRT-PCR); as shown in **Figure 3B**, Fabp4 and PPARγ mRNA levels were significantly upregulated in adipocytes compared with preadipocytes. Then, we built the IR adipocyte model in differentiated adipocytes as described. There was no difference of Fabp4 and PPARγ mRNAs between control adipocytes and IR adipocytes (**Figure 3C**). To validate the IR adipocyte model, we examined glucose uptake ability and triglyceride content. **Figure 3D** shows that the 2-DG uptake rate was reduced in the IR adipocyte model. Moreover, triglyceride excessive accumulation also appeared in IR adipocytes (**Figure 3E**). Cellular membrane-located GLUT4 levels were decreased in IR adipocytes as revealed by immunofluorescence (IF) staining and immunoblotting (**Figures 3F, G**). Insulin resistance is a pathological phenomenon in which insulin cannot inhibit the expression of gluconeogenic genes, which is mainly mediated through the Akt pathway (27). Thus, we examined Akt and p-Akt protein contents in control or IR adipocytes; as shown in **Figure 3H**, the ratio of p-Akt/Akt decreased in IR adipocytes. Moreover, TNF-α and IL-6 mRNA expression and secretion levels were upregulated in IR adipocytes (**Figures 3I, J**), whereas the protein levels of DMRT2 were decreased in IR adipocytes (**Figure 3K**).

### *In-Vitro* Effects of DMRT2 on IR Adipocytes

**Figure 4:**
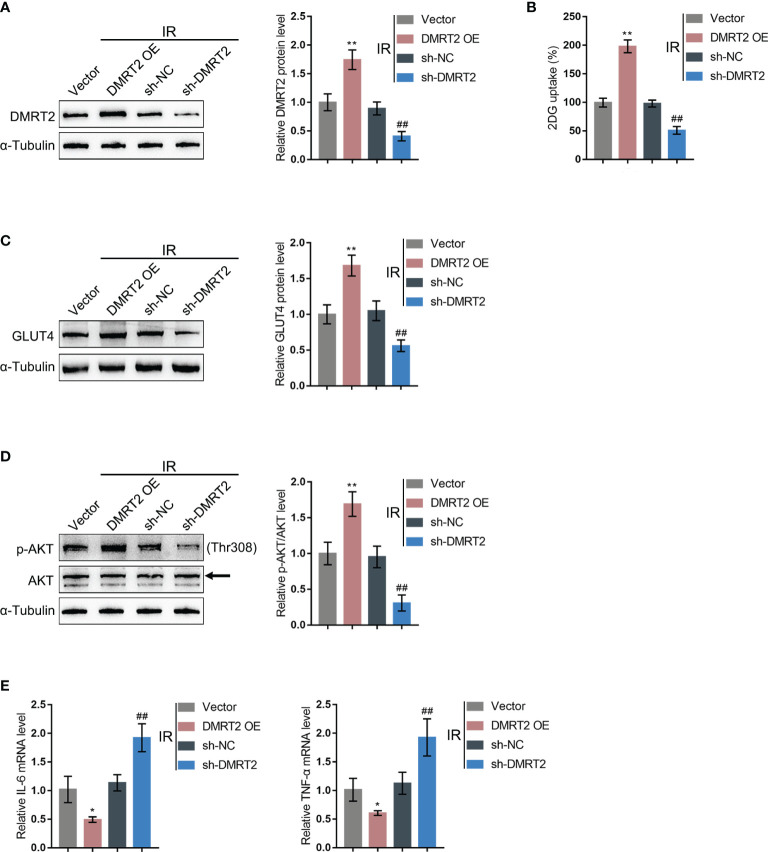
*In-vitro* effects of DMRT2 on IR adipocytes. **(A)** DMRT2 overexpression or knockdown was achieved in adipocytes by transducing DMRT2-overexpressing vector (DMRT2 OE) or small interference RNA for DMRT2 (sh-DMRT2). The overexpression or knockdown of DMRT2 was confirmed using immunoblotting. Then, IR adipocytes were transfected with DMRT2 OE or sh-DMRT2 and examined for glucose uptake ability **(B)**; protein levels of GLUT4 by immunoblotting **(C)**; the protein levels of Akt and p-Akt by immunoblotting **(D)**; and the mRNA expression of TNF-α and IL-6 in IR adipocytes by qRT-PCR **(E)**. *P < 0.05, **P < 0.01, compared with the vector group; ^##^ *P* < 0.01, compared sh-NC with the sh-DMRT2 group.

To confirm the specific effects of DMRT2 on IR adipocytes, we achieved DMRT2 overexpression or knockdown in IR adipocytes by transducing DMRT2-overexpressing vector (DMRT2 OE) or small interference RNA for DMRT2 (sh-DMRT2). The overexpression or knockdown of DMRT2 was confirmed using immunoblotting (**Figure 4A**). Then, IR adipocytes were transfected with DMRT2 OE or sh-DMRT2 and examined for related indexes. DMRT2 overexpression increased but DMRT2 knockdown decreased the glucose uptake (**Figure 4B**). DMRT2 overexpression increased the protein levels of GLUT4 (**Figure 4C**) and the ratio of p-Akt/Akt (**Figure 4D**), whereas DMRT2 knockdown exerted opposite effects. Consistently, DMRT2 overexpression downregulated but DMRT2 knockdown upregulated TNF-α and IL-6 mRNA expression in IR adipocytes (**Figure 4E**). Moreover, in normal adipocytes, DMRT2 overexpression also could increase glucose uptake, GLUT4 expression, and the ratio of p-Akt/Akt and reduce the mRNA level of TNF-α and IL-6. DMRT2 knockdown exerted opposite effects on those factors (**Figure S4**).

### DMRT2 Directly Interacts With FXR

**Figure 5:**
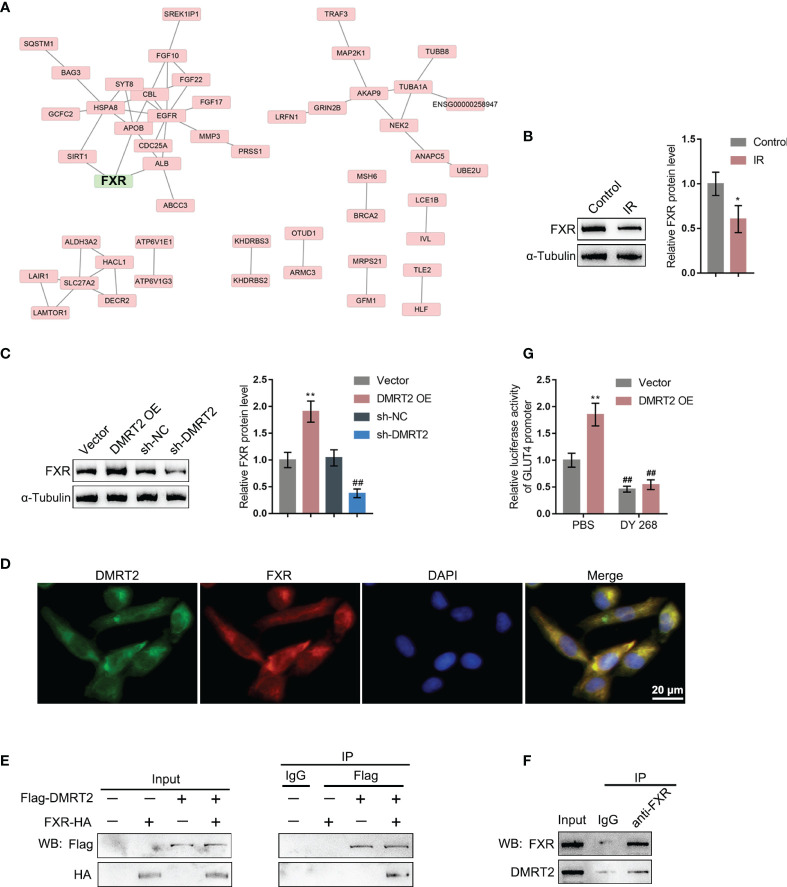
DMRT2 directly interacts with FXR. **(A)** Protein–protein interaction analysis showing proteins that might interact with DMRT2. **(B)** The protein levels of FXR in IR adipocytes and IF adipocytes were examined using immunoblotting. **(C)** IR adipocytes were transfected with DMRT2 OE or sh-DMRT2 and examined for the protein levels of FXR by immunoblotting. **(D)** The interaction between DMRT2 and FXR was examined using IF staining. Green fluorescence indicated DMRT2. Red fluorescence indicated FXR. **(E, F)** The interaction between DMRT2 and FXR was examined using the co-IP assay. **(G)** 3T3-L1 cells were co-transfected with DMRT2 overexpression vector and GLUT4 promoter luciferase reporter vector. Then, the luciferase activity was measured. *P < 0.05, **P < 0.01, compared with the control or vector group; ^##^ *P* < 0.01, compared sh-NC with the sh-DMRT2 group.

To further investigate the underlying mechanism, we performed protein–protein interaction analysis searching for proteins that might interact with DMRT2 (**Figure 5A**); notably, FXR, which is closely related to glucose and lipid metabolism, was also included. Next, FXR protein contents within control adipocytes and IR adipocytes were determined. **Figure 5B** shows that FXR protein levels were significantly decreased in IR adipocytes. Moreover, in IR adipocytes, DMRT2 overexpression increased but DMRT2 knockdown decreased FXR protein levels (**Figure 5C**). The IF staining results showed that DMRT2 protein may collocate with FXR protein (**Figure 5D**). To further confirm the interaction between DMRT2 and FXR, we performed the co-IP assay and constructed Flag-DMRT2 and FXR-HA vectors and co-transfected these vectors into adipocytes. As shown by immunoblotting, DMRT2 and FXR could interact with each other (**Figure 5E**). The endogenous FXR and DMRT2 co-IP also confirmed this binding interaction (**Figure 5F**). Moreover, the luciferase assay confirmed that DMRT2 overexpression increased the GLUT4 promoter activity. The FXR-specific inhibitor DY268 (5 μM for 24 h) effectively reduced the activity both in the vector and DMRT2 overexpression groups (**Figure 5G**). These results indicated that DMRT2 could increase the FXR transcription activity in GLUT4 transcription.

### Dynamic Effects of DMRT2 and FXR on Adipocyte Resistance to Insulin

**Figure 6:**
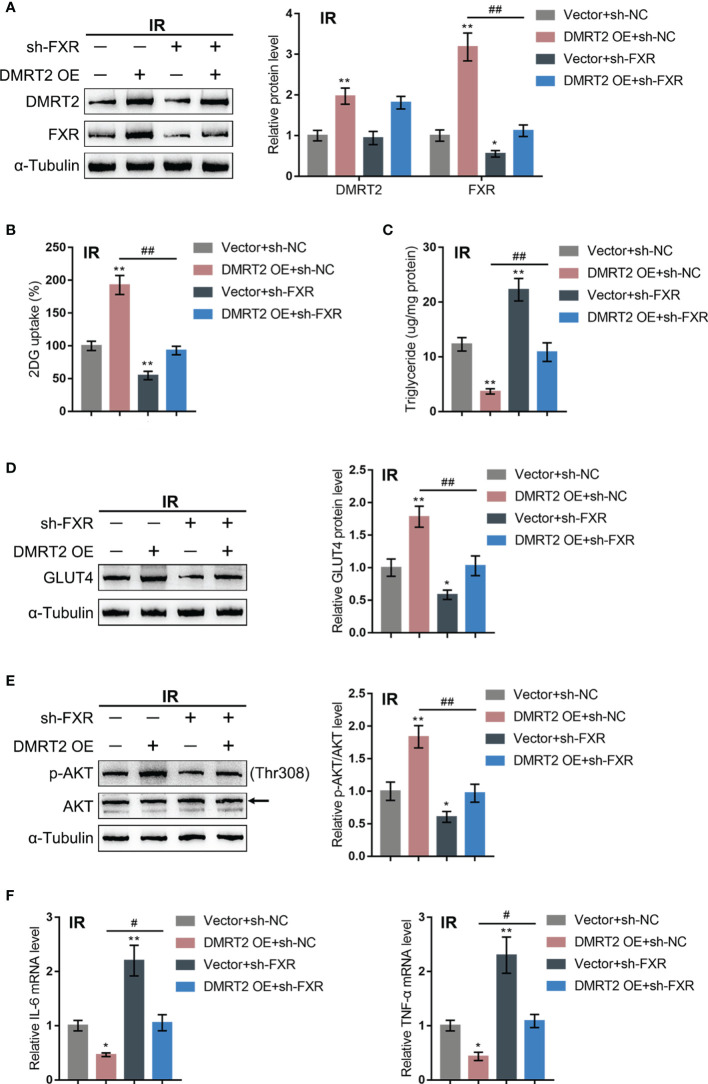
Dynamic effects of DMRT2 and FXR on adipocyte resistance to insulin. IR adipocytes were co-transfected with DMRT2 OE and sh-FXR and examined for the protein levels of DMRT2 and FXR by immunoblotting **(A)**; glucose uptake ability **(B)**, triglyceride content **(C)**, and the protein levels of GLUT4 by immunoblotting **(D)**; the protein levels of Akt and p-Akt by immunoblotting **(E)**; and the mRNA expression of TNF-α and IL-6 in control or IR adipocytes by qRT-PCR **(F)**. *P < 0.05, **P < 0.01, compared with the Vector+sh-NC group; ^#^ *P* < 0.05, ^##^ *P* < 0.01, compared Vector+sh-FXR with the DMRT2 OE+sh-FXR group.

After confirming the interaction between DMRT2 and FXR, we examined the dynamic effects of DMRT2 and FXR on the resistance of adipocytes to insulin. IR adipocytes were co-transfected with DMRT2 OE and sh-FXR. DMRT2 OE caused increases in both DMRT2 and FXR protein levels, whereas sh-FXR caused no change in DMRT2 and decreased FXR; the promotive effects of DMRT2 OE on FXR were reversed by sh-FXR (**Figure 6A**). Thus, DMRT2 could regulate FXR levels, and FXR is downstream of DMRT2. DMRT2 OE increased but sh-FXR decreased glucose uptake; the effects of DMRT2 OE on glucose uptake were reversed by sh-FXR (**Figure 6B**). In contrast, the triglyceride content was decreased by DMRT2 overexpression and increased by sh-FXR (**Figure 6C**). DMRT2 OE increased but sh-FXR decreased the protein levels of GLUT4 (**Figure 6D**) and the ratio of p-Akt/Akt (**Figure 6E**); the effects of DMRT2 OE were reversed by sh-FXR (**Figure 6E**). Finally, DMRT2 OE downregulated but sh-FXR upregulated TNF-α and IL-6 mRNA expression; the effects of DMRT2 OE on the mRNA expression levels of TNF-α and IL-6 were reversed by sh-FXR (**Figure 6F**). Moreover, in normal adipocytes, DMRT2 OE-induced upregulation of glucose uptake, GLUT4 expression, and the ratio of p-Akt/Akt and downregulation of mRNA expression levels of TNF-α and IL-6 could be reversed by sh-FXR (**Figure S5**).

## Discussion

Herein, the expression level of DMRT2 was shown to be downregulated in adipose tissues from insulin-resistant subjects through bioinformatics analysis and in an insulin-resistant mouse model through experimental analysis. DMRT2 overexpression significantly improved HDF-induced insulin resistance and inflammation in mice. Moreover, in control and insulin-resistant differentiated mouse 3T3-L1 adipocytes, DMRT2 overexpression improved but DMRT2 knockdown enhanced the insulin resistance of 3T3-L1 adipocytes. DMRT2 interacted with FXR and positively regulated FXR level and transcription activity. In differentiated mouse 3T3-L1 adipocytes, FXR knockdown enhanced insulin resistance and attenuated the effects of DMRT2 overexpression upon 3T3-L1 adipocyte insulin resistance.

As a critical factor resulting in insulin resistance, obesity is often accompanied by adipocyte enlargement, macrophage infiltration, excessive secretion of triglycerides in adipose tissues, and many other alterations (28). It is now well accepted that in homogeneous C57BL/6J mice, HFD feeding leads to resistance to insulin (29). Consistent with these previous findings, we reported that exposure to an HFD for 12 weeks elicits glucose metabolic impairments, manifested as altered glucose tolerance and insulin tolerance, increased epididymal fat weight, and reduced brown adipocytes. Macrophage infiltration and pro-inflammatory cytokine levels were also increased in epididymal white adipose of HFD mice. After overexpressing DMRT2, blood sugar level, resistance to insulin, and inflammation in mice were improved. Moreover, DMRT2 overexpression also reduced TNF-α and IL-6 levels, suggesting that DMRT2 overexpression promoted HFD-caused resistance to insulin in mice from multiple aspects.

To further confirm the improving effects of DMRT2 overexpression on HFD-induced insulin resistance of mice, we established the insulin resistance model of adipocytes by the dexamethasone induction method. In insulin-resistant adipocytes, the cellular glucose uptake and membrane GLUT4 expression were both significantly inhibited. After overexpressing DMRT2, GLUT4 levels were increased and TNF-α and IL-6 levels were decreased, suggesting that overexpression of DMRT2 can enhance the sensitivity of adipocytes to insulin. Glucose transporters 4 (GLUT4) is mainly expressed in adipose tissue, and it exerts a crucial effect on maintaining the steady-state of glucose metabolism in the body (30, 31). More importantly, the decrease in the expression or activity of GLUT4 is an important molecular basis that leads to a decrease in glucose uptake and utilization by adipocytes, which in turn induces insulin resistance (30, 31). Considering the findings in the present study, DMRT2 may participate in insulin resistance by regulating the metabolic process of adipocytes.

As for the underlying molecular mechanism, we performed protein–protein interaction to retrieve proteins that may interact with DMRT2, and PDLIM7, HNRNPUL2, ACTB, RPL7, and FXR were found. The bile-acid-activated nuclear receptor, farnesoid X receptor (FXR), is a ligand-dependent transcription factor regulating the transcription of some key metabolic enzymes in the processes of bile acid metabolism and glucose and lipid metabolism (32, 33). Studies on FXR and fat metabolism show that FXR^−/−^ mice have significantly increased liver glycolysis and lipogenesis gene expression when fasting and then eating, indicating that FXR deficiency can enhance glycolysis pathways and provide a large number of substrates for lipogenesis (34). The use of the FXR agonist BAR502 can significantly reduce the insulin resistance of non-alcoholic steatohepatitis model mice, thereby promoting white fat browning and reversing liver steatosis (35). FXR agonist administration in the rabbit metabolic syndrome model can reduce the insulin resistance of adipose tissue (36). In 3T3-L1 cells, the FXR agonist increased Akt phosphorylation and glucose uptake (37). Moreover, Shen et al. reported that in human and mouse adipocytes, FXR promoted GLUT4 expression *via* FXR response element in GLUT4 promoter (22). In this study, we found that DMRT2 could bind to FXR and promote its transcription activity on GLUT4. Knockdown of FXR could reverse DMRT2 overexpression effects on inflammation and glucose uptake, triglyceride accumulation, GLUT4 expression, and AKT phosphorylation. The above studies show that FXR exerts a critical effect on regulating the resistance of adipose tissues to insulin, and FXR is an important target protein of DMRT2.

In conclusion, we identify the downregulation of DMRT2 in the insulin-resistant mouse model and cell model. DMRT2 reduced insulin resistance *via* modulating FXR expression and transcription activity in adipocytes. However, the effects of DMRT2 on hepatic bile acid and lipid metabolism need to be further investigated.

## Data Availability Statement

The original contributions presented in the study are included in the article/**Supplementary Material**. Further inquiries can be directed to the corresponding authors.

## Ethics Statement

All experimental procedures were in compliance with the National Institutes of Health guidelines and were approved by the local Animal Care and Use Committee of the People’s Hospital of Xinjiang Uygur Autonomous Region.

## Author Contributions

JT designed the analysis and wrote the paper. X-LY and Y-JY collected the data. XS, JL, and P-PG contributed data or analysis tools. ZW, Y-TM, and G-QL were responsible for the project administration. All authors contributed to the article and approved the submitted version.

## Funding

This study was supported by Natural Science Foundation of China grant (82060164) and Xinjiang Uygur Autonomous Region Regional Collaborative Innovation Project (Science and Technology Aid Xinjiang Program) (2017E0270).

## Conflict of Interest

The authors declare that the research was conducted in the absence of any commercial or financial relationships that could be construed as a potential conflict of interest.

## Publisher’s Note

All claims expressed in this article are solely those of the authors and do not necessarily represent those of their affiliated organizations, or those of the publisher, the editors and the reviewers. Any product that may be evaluated in this article, or claim that may be made by its manufacturer, is not guaranteed or endorsed by the publisher.

## Supplementary Material

Supplementary Figure 1 Expression of DMRT2 in insulin-resistance **(A)** Screening process of DMRT2 **(B)** DMRT2 expression in subcutaneous and omental abdominal adipose tissues from non-obese and obese subjects according to GSE15524. **(C)** DMRT2 expression in subcutaneous adipose tissue from lean and obese subjects according to GSE12050. **(D)** DMRT2 expression in insulin-resistant or insulin-sensitive adipose tissues according to GSE15773. **(E)** The list of genes positively correlated with DMRT2 expression in 1794 sets of gene microarray data from different tissues analyzed using the MEM database matrix.

Supplementary Figure 2 The protein level of DMRT2 in subcutaneous adipose tissue

Supplementary Figure 3 DMRT2 increased the expression of BSEP, SHP and decreased CYP7A1 expression in liver tissues of insulin-resistant mouse model. **(A, B)** The protein level of BSEP, SHP, and CYP7A1 were examined using immunobloting and IHC staining. **P<0.01, compared with the Normal group; ^#^P < 0.05, ^##^P < 0.01, compared Lv-Vector+HFD with Lv- DMRT2 OE+HFD group.

Supplementary Figure 4
*In vitro* effects of DMRT2 on control adipocytes. **(A)** DMRT2 overexpression or knockdown was achieved in control adipocytes by transducing DMRT2-overexpressing vector (DMRT2 OE) or small interferences RNA for DMRT2 (sh-DMRT2). The overexpression or knockdown of DMRT2 was confirmed using Immunoblotting. Then, control adipocytes were transfected with DMRT2 OE or sh-DMRT2 and examined for glucose uptake ability **(B)**; protein levels of GLUT4 by Immunoblotting **(C)**; the protein levels of Akt and p-Akt by Immunoblotting **(D)**; the mRNA expression of TNF-α and IL-6 in control adipocytes by qRT-PCR **(E)**. *P < 0.05, **P < 0.01, compared with the Vector group; ^##^P < 0.01, compared sh-NC with sh- DMRT2 group.

Supplementary Figure 5 Dynamic effects of DMRT2 and FXR on control adipocyte. Control adipocytes were co-transfected with DMRT2 OE and sh-FXR and examined for the protein levels of DMRT2 and FXR by Immunoblotting **(A)**; glucose uptake ability **(B)**; triglyceride content **(C)**; the protein levels of GLUT4 by Immunoblotting **(D)**; the protein levels of Akt and p-Akt by Immunoblotting **(E)**; the mRNA expression of TNF-α and IL-6 in control adipocytes by qRT-PCR **(F)**. *P < 0.05, **P < 0.01, compared with the Vector+sh-NC group; ^#^P < 0.05, ^##^P < 0.01, compared Vector+sh-FXR with DMRT2 OE+ sh-FXR group.
