# Supplementary figures and images for: DMRT2 Interacts With FXR and Improves Insulin Resistance in Adipocytes and a Mouse Model
Source: Front Endocrinol (Lausanne). 2022 Feb 17;12:723623. doi: 10.3389/fendo.2021.723623 (PMC8891600; doi:10.3389/fendo.2021.723623)

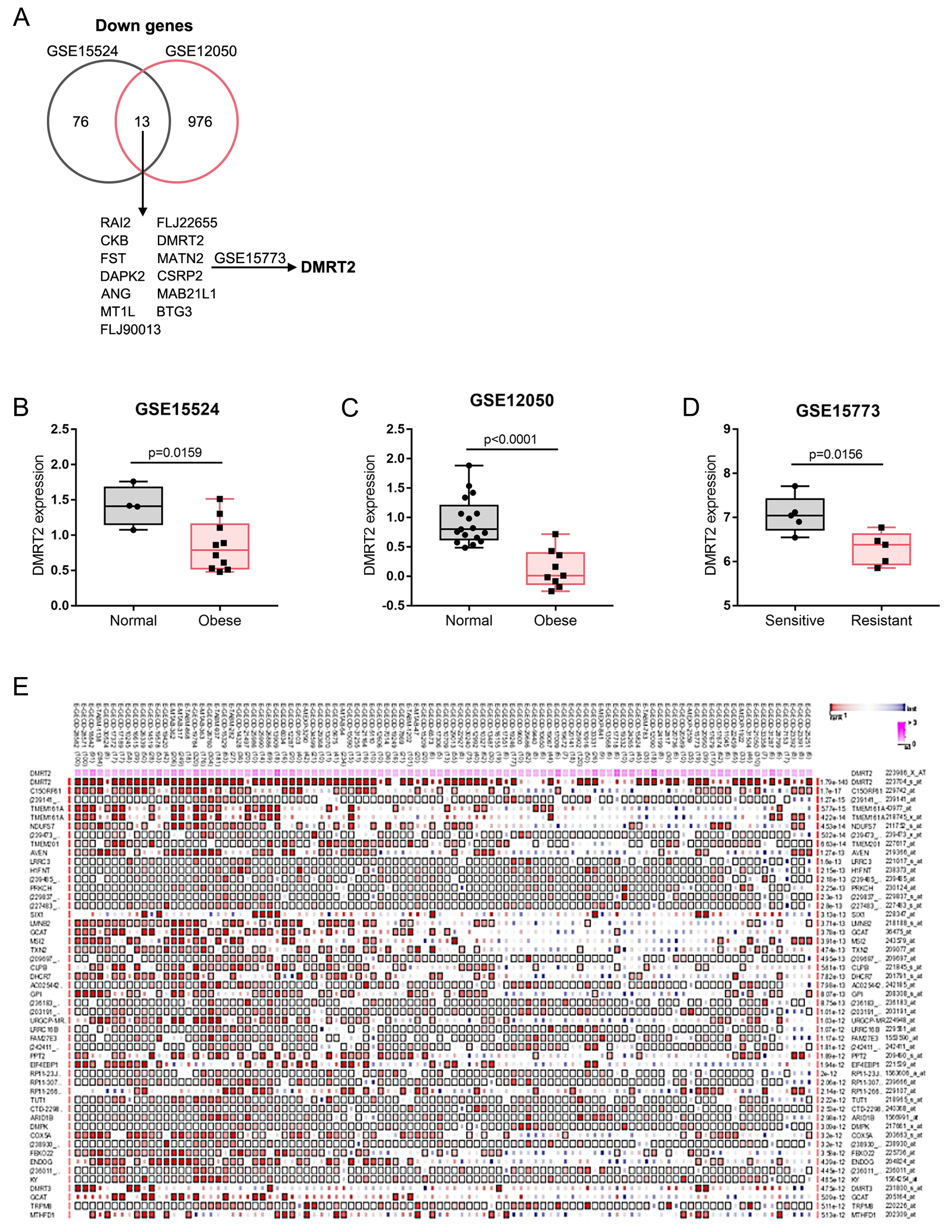

Supplement: Supplementary Figure 1 — Expression of DMRT2 in insulin-resistance (A) Screening process of DMRT2 (B) DMRT2 expression in subcutaneous and omental abdominal adipose tissues from non-obese and obese subjects according to GSE15524. (C) DMRT2 expression in subcutaneous adipose tissue from lean and obese subjects according to GSE12050. (D) DMRT2 expression in insulin-resistant or insulin-sensitive adipose tissues according to GSE15773. (E) The list of genes positively correlated with DMRT2 expression in 1794 sets of gene microarray data from different tissues analyzed using the MEM database matrix. [file Image_1.tif]

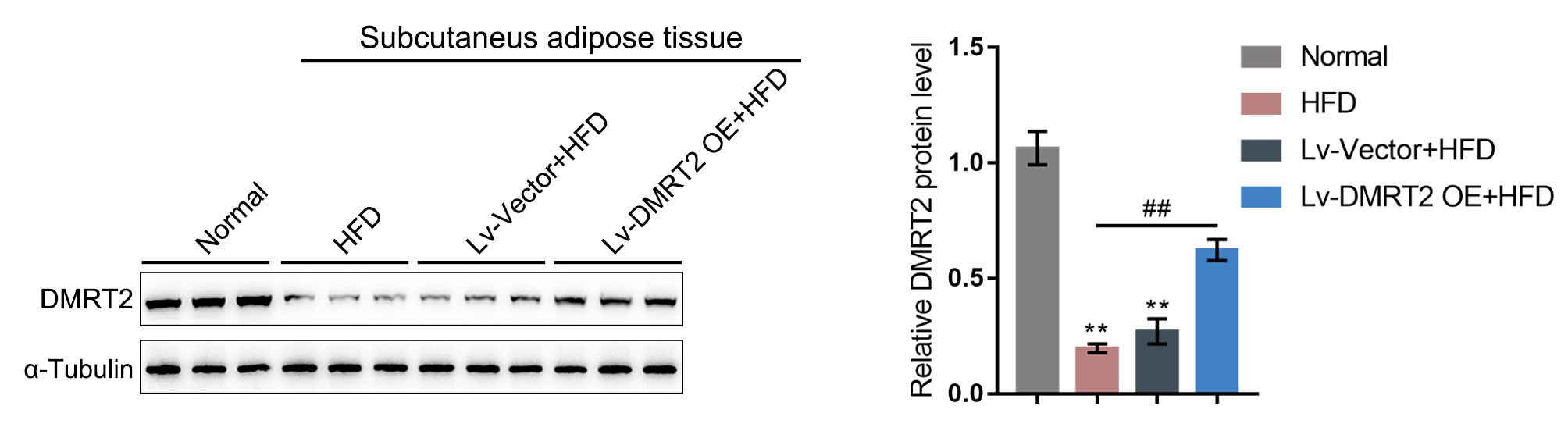

Supplement: Supplementary Figure 2 — The protein level of DMRT2 in subcutaneous adipose tissue [file Image_2.tif]

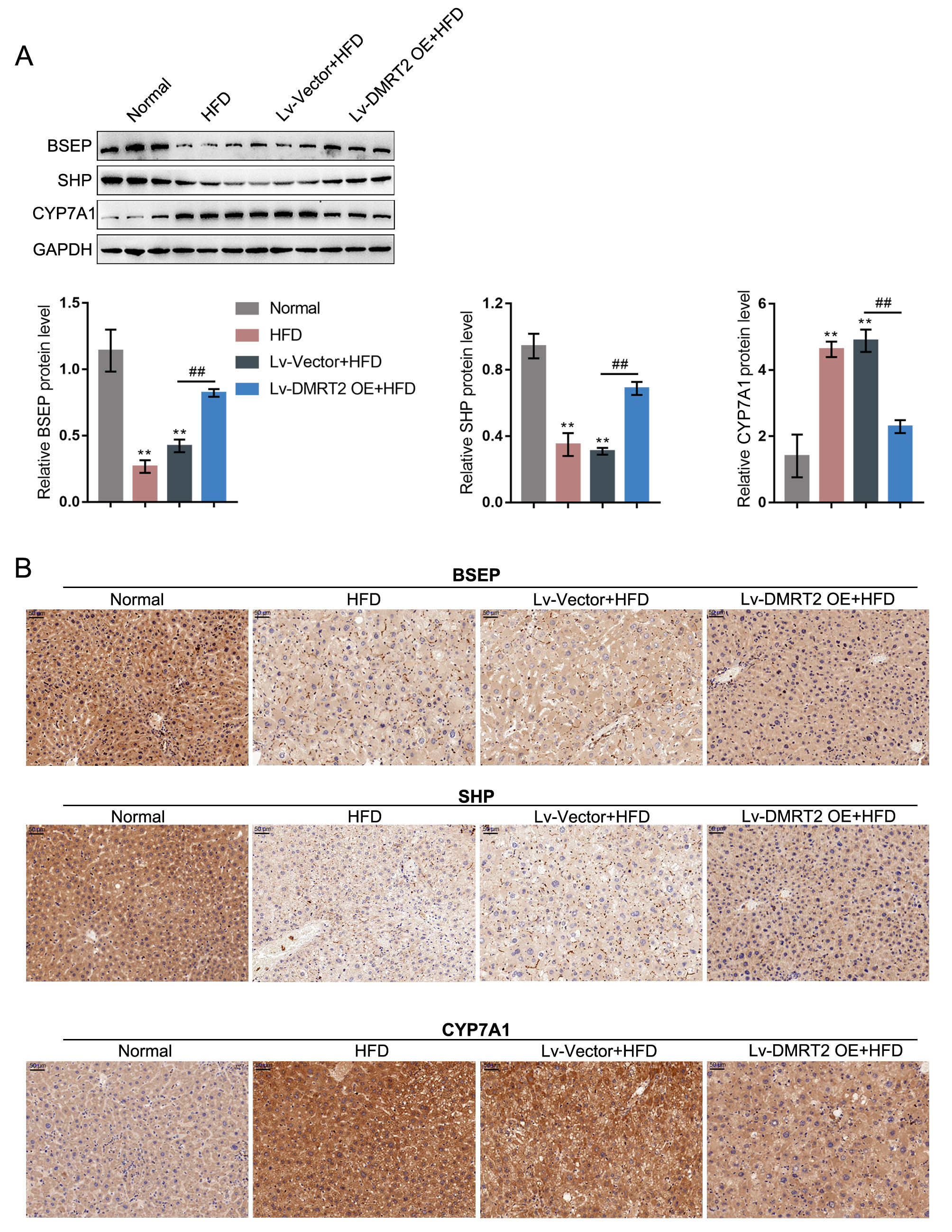

Supplement: Supplementary Figure 3 — DMRT2 increased the expression of BSEP, SHP and decreased CYP7A1 expression in liver tissues of insulin-resistant mouse model. (A, B) The protein level of BSEP, SHP, and CYP7A1 were examined using immunobloting and IHC staining. **P<0.01, compared with the Normal group; #P < 0.05, ##P < 0.01, compared Lv-Vector+HFD with Lv- DMRT2 OE+HFD group. [file Image_3.tif]

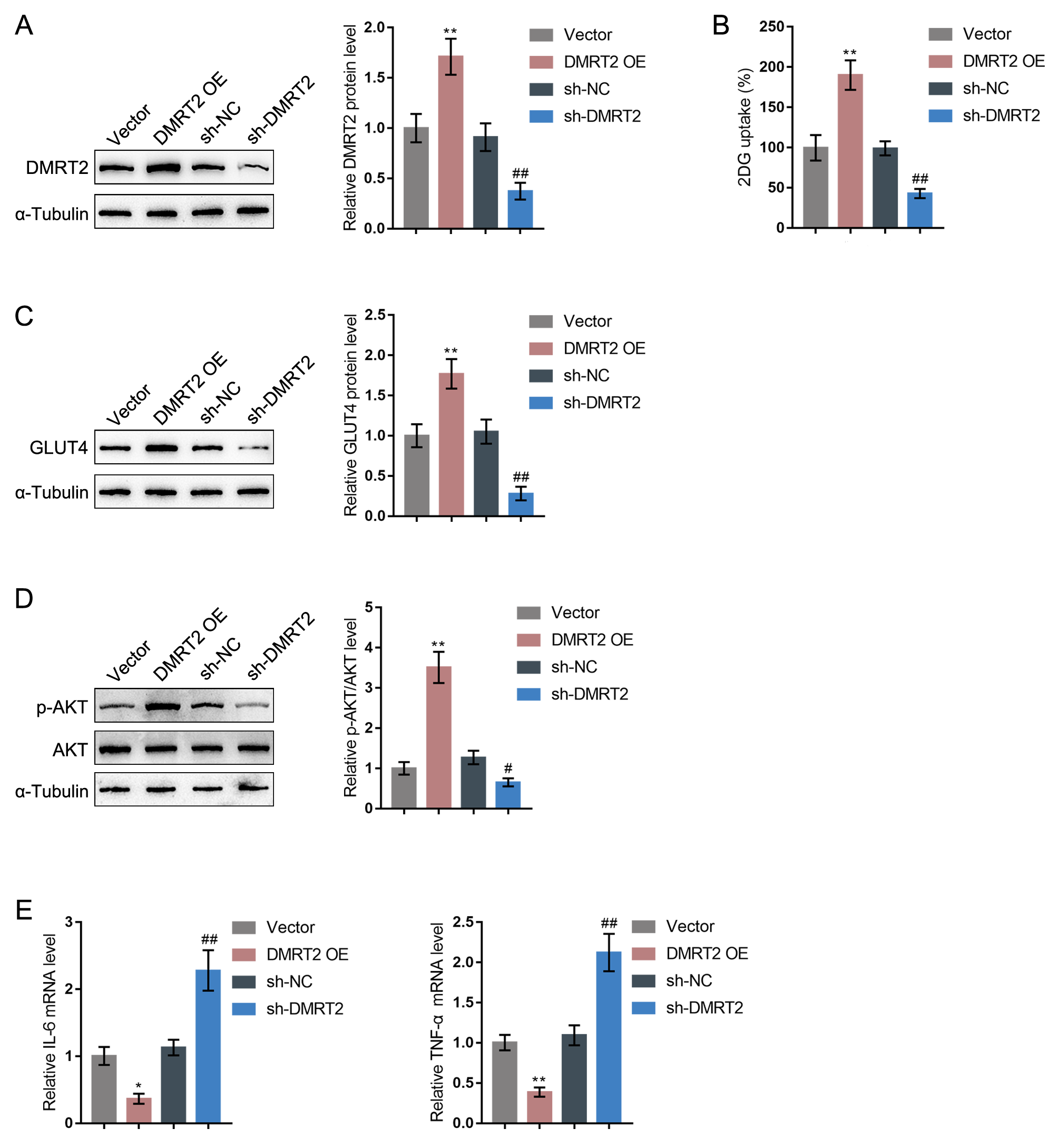

Supplement: Supplementary Figure 4 — In vitro effects of DMRT2 on control adipocytes. (A) DMRT2 overexpression or knockdown was achieved in control adipocytes by transducing DMRT2-overexpressing vector (DMRT2 OE) or small interferences RNA for DMRT2 (sh-DMRT2). The overexpression or knockdown of DMRT2 was confirmed using Immunoblotting. Then, control adipocytes were transfected with DMRT2 OE or sh-DMRT2 and examined for glucose uptake ability (B); protein levels of GLUT4 by Immunoblotting (C); the protein levels of Akt and p-Akt by Immunoblotting (D); the mRNA expression of TNF-α and IL-6 in control adipocytes by qRT-PCR (E). *P < 0.05, **P < 0.01, compared with the Vector group; ##P < 0.01, compared sh-NC with sh- DMRT2 group. [file Image_4.tif]

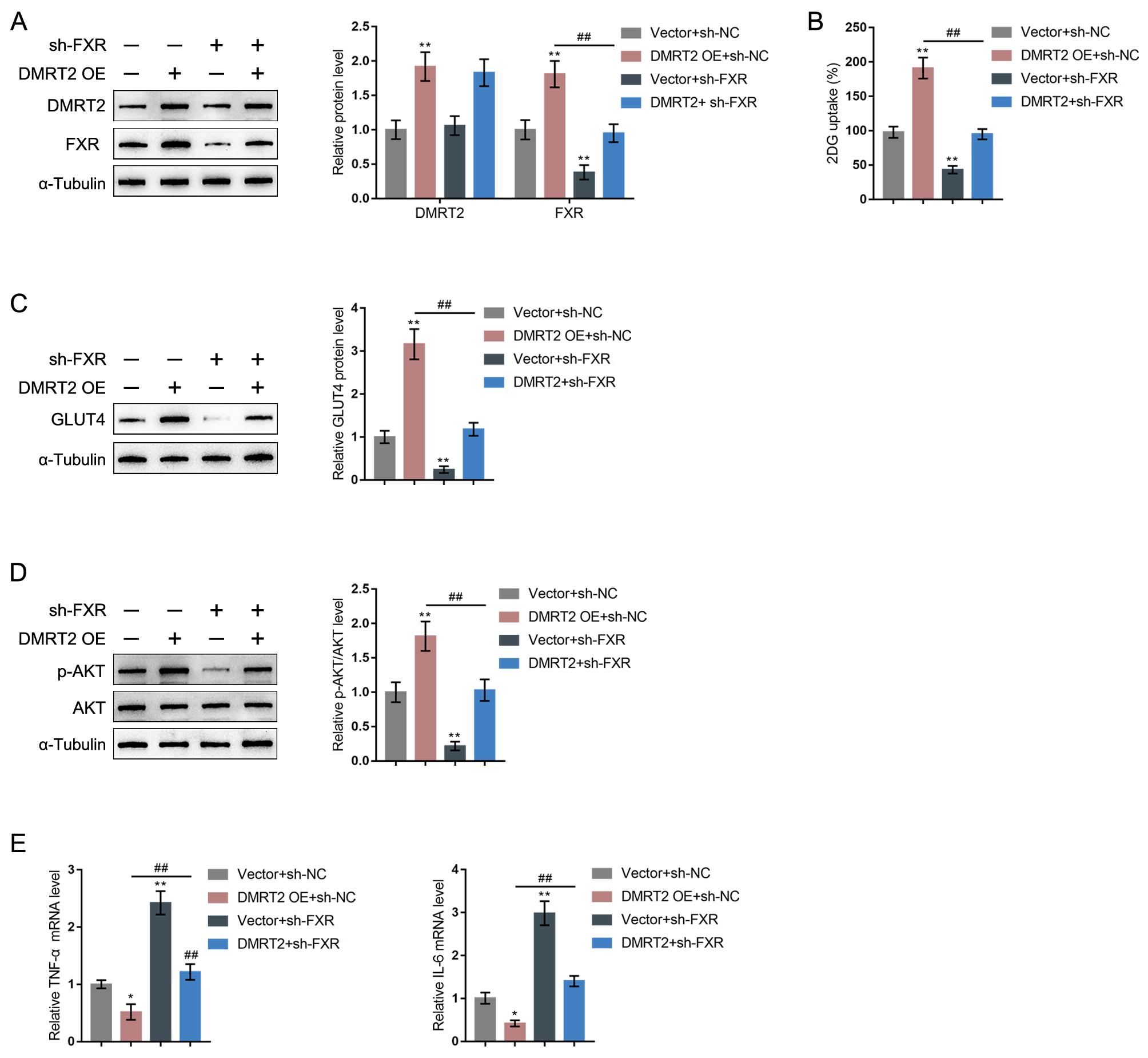

Supplement: Supplementary Figure 5 — Dynamic effects of DMRT2 and FXR on control adipocyte. Control adipocytes were co-transfected with DMRT2 OE and sh-FXR and examined for the protein levels of DMRT2 and FXR by Immunoblotting (A); glucose uptake ability (B); triglyceride content (C); the protein levels of GLUT4 by Immunoblotting (D); the protein levels of Akt and p-Akt by Immunoblotting (E); the mRNA expression of TNF-α and IL-6 in control adipocytes by qRT-PCR (F). *P < 0.05, **P < 0.01, compared with the Vector+sh-NC group; #P < 0.05, ##P < 0.01, compared Vector+sh-FXR with DMRT2 OE+ sh-FXR group. [file Image_5.tif]
