# Supplementary material for: DMRT2 Interacts With FXR and Improves Insulin Resistance in Adipocytes and a Mouse Model
Source: Front Endocrinol (Lausanne). 2022 Feb 17;12:723623. doi: 10.3389/fendo.2021.723623 (PMC8891600; doi:10.3389/fendo.2021.723623)
Supplement: Supplementary file 6 [file Table_1.doc]

**Table S1 The primer sequence for RT-PCR**

| RT-PCR | Forward | Reverse |
| --- | --- | --- |
| Il-6 | TAGTCCTTCCTACCCCAATTTCC | TTGGTCCTTAGCCACTCCTTC |
| TNF-α | CCCTCACACTCAGATCATCTTCT | GCTACGACGTGGGCTACAG |
| DMRT2 | TACCTCCGCCTGTCAGTGA | GTTGGGCAGGAATAAGGCAG |
| Fabp4 | AAGGTGAAGAGCATCATAACCCT | TCACGCCTTTCATAACACATTCC |
| PPARγ | CTCCAAGAATACCAAAGTGCGA | GCCTGATGCTTTATCCCCACA |
| ACTB | GGCTGTATTCCCCTCCATCG | CCAGTTGGTAACAATGCCATGT |
